# Overexpression of lncRNA SNGH3 Predicts Unfavorable Prognosis and Clinical Outcomes in Human Cancers: Evidence from a Meta-Analysis

**DOI:** 10.1155/2020/7974034

**Published:** 2020-06-25

**Authors:** Yaofei Jiang, Lulu Le

**Affiliations:** ^1^The Second Affiliated Hospital of Nanchang University, Nanchang, Jiangxi Province 330000, China; ^2^Department of Radiation and Medical Oncology, Zhongnan Hospital, Wuhan University, Wuhan, Hubei Province 430000, China

## Abstract

Long noncoding RNAs (lncRNAs) have been confirmed to play a crucial role in human disease, especially in tumor development and progression. Small nucleolar RNA host gene (SNHG3), a newly identified lncRNA, has been found dysregulated in various cancers. Nevertheless, the results remain controversial. Thus, we aim to analyze the comprehensive data to elaborate the association between SNHG3 expression and clinical outcomes in multiple cancers. We searched PubMed, Web of Science, Cochrane Library, Embase, and MEDLINE database to identify eligible articles. STATA software was applied to calculate the hazard ratio (HR) and odds ratio (OR) with 95% confidence interval (95% CI) for survival outcomes and clinical parameters, respectively. Besides, the data from The Cancer Genome Atlas (TCGA) dataset was extracted to verify the results in our meta-analysis. There were thirteen studies totaling 919 cancer patients involved in this meta-analysis. The results demonstrated that high SNHG3 expression was significantly associated with poor overall survival (OS) (HR = 2.53, 95% CI: 1.94-3.31) in cancers, disease-free survival (DFS) (HR = 3.89, 95% CI: 1.34-11.3), and recurrence-free survival (RFS) (HR = 2.42, 95% CI: 1.14-5.15) in hepatocellular carcinoma. Analysis stratified by analysis method, sample size, follow-up time, and cancer type further verified the prognostic value of SNHG3. Additionally, patients with high SNHG3 expression tended to have more advanced clinical stage, higher histological grade, earlier distant metastasis, and earlier lymph node metastasis. Excavation of TCGA dataset valuated that SNHG3 was upregulated in various cancers and predicted worse OS and DFS. Overexpressed SNHG3 was strongly associated with poor survival and clinical outcomes in human cancers and therefore can serve as a promising biomarker for predicting patients' prognosis.

## 1. Introduction

Nowadays, cancer is the most prevalent cause of death and continues to be a serious public health problem with increasing morbidity and mortality [1, 2]. It was reported that about 9.6 million people died of cancers and more than 18.1 million people diagnosed with cancers in 2018 worldwide [3]. Although clinical treatment including surgery, radiotherapy, and chemotherapy, as well as targeted therapy and immunotherapy improved dramatically in the last century, patients' 5-year survival rate is still low and remains to be enhanced due to malignant progression and deficiencies in early diagnosis and treatment target [4–6]. On account of early diagnosis and treatment that can greatly improve cancer patients' survival, it is urgent to discover new biomarkers for cancer patients [7].

Long noncoding RNAs (lncRNAs) are mRNA-like transcripts, which consist of more than 200 nucleotides and lack protein-coding ability [8, 9]. Considered genomic “noise” initially, lncRNAs get increasing attention due to their newly found role in various diseases and cellular activity nowadays [10, 11]. Especially in tumor, more and more studies confirm that lncRNAs involve in tumorigenesis and metastasis through various mechanisms, such as sponging miRNAs, epigenetic regulation, translation regulation, cell differentiation regulation, and therapy resistance [12–14]. The expression and role of different lncRNAs may vary greatly. While some lncRNAs express as tumor suppressor genes, like GAS5 and ANRIL, more lncRNAs functioned as oncogenes, such as NKILA and FAL1 [15–18]. Notably, these lncRNAs are expected to be biomarkers and therapeutic targets for cancers thereby.

Small nucleolar RNA host gene 3 (SNHG3) is a newly identified lncRNA with abnormal expression in various cancers. Previous studies reported that SNHG3 is upregulated in cancers and may act as an oncogene in tumor prognosis, including osteosarcoma [19], glioma [20], ovarian cancers [21], breast cancer [22], and hepatocellular carcinoma [23]. Overexpression of SNHG3 in these was usually associated with poor prognosis and clinical features, like advanced clinical stage, earlier distant metastasis, and tumor size. Furthermore, SNHG3 involved in the pathologic process of tumor, including cell proliferation, migration, EMT, and apoptosis [24]. Collectively, SNHG3 may be a promising biomarker for diagnosis and target for therapy in various cancers. However, the results remain to be confirmed due to contentious outcomes and small sample size in individual studies. Thus, we preformed this meta-analysis and review, for the first time, to identify the clinical role of SNHG3 in human cancers and explore its functions.

## 2. Materials and Methods

The present meta-analysis was constructed and reported according to the PRISMA checklist [25].

### 2.1. Information Source and Searching Strategy

We searched many databases, including PubMed, Web of Science, Cochrane Library, Embase, and MEDLINE, by retrieved keywords in them from March 1, 2020, to March 5, 2020, to collect all eligible studies for this meta-analysis. We used the following keywords and MeSH terms: (“small nucleolar RNA host gene 3” or “SNHG3”) and (“cancer” or “carcinoma” or “neoplasm” or “tumor”) and (“prognosis” or “clinical outcome” or “pathological feature” or “survival”). The search syntax is shown in Table S1. Additional records were identified through searching for references of these retrieval studies.

### 2.2. Eligibility Criteria

Studies meeting the following criteria were enrolled in this meta-analysis: (a) reported the expression of SNHG3 in tumor tissues; (b) valuable data in regard to association between SNHG3 expression and clinical parameters; (c) providing hazard ratio (HR) with 95% confidence intervals (CI) for survival outcomes directly; (d) sufficient data to calculate the HRs and CIs for survival outcomes; and (e) written in English. Studies meeting one or more of the following criteria were excluded: (a) duplicate publications; (b) based on other diseases but not cancers; (c) animal studies; (d) not available date for survival outcomes and clinical parameters; and (e) letters, case reports, expert opinions, and reviews.

### 2.3. Data Extraction and Quality Valuation

Two authors (YJ and LL) recorded data and corresponding basic information from included studies and evaluated the quality of them independently, including surname of first author, publication year, country of the study carried out at, cancer type, sample size, specimen, detection method, cutoff value, follow-up time, and analysis method. Any disagreements were resolved by a doctor who is professional in this area. Meanwhile, clinical parameters including age, gender, clinical stage, tumor size, lymph node metastasis, distant metastasis, and histological grade were also extracted. For patients' survival outcomes, including overall survival (OS), metastasis-free survival (MFS), disease-free survival (DFS), and recurrence-free survival (RFS), we recorded HRs and 95% CIs from studies provided to them directly or using Engauge Digitizer version 4.1 to extract the data from those studies that provided Kaplan-Meier curves only indirectly. The Newcastle-Ottawa Scale (NOS) was used to evaluate the quality of included studies, and studies with NOS score 7-9 were considered of high quality [26].

### 2.4. Validation by Using Data from TCGA Dataset

Gene Expression Profiling Interactive Analysis (GEPIA) is a newly developed interactive web aiming at analyzing the RNA sequencing expression data from The Cancer Genome Atlas (TCGA) dataset [27]. It was used to explore the SNHG3 expression level in tumor and normal tissues in different kinds of cancers. Kaplan-Meier method and logrank test were applied to calculate the survival analysis to validate the association between SNHG3 expression and OS and DFS.

### 2.5. Statistical Analysis

STATA software (version 12.0; StataCorp LLC, College Station, Texas) was applied to analyze the HRs and 95% CIs for survival outcomes and ORs and 95% CIs for clinical parameters. Chi squared-based *Q* test and *I*^2^ statistics were performed to identify the heterogeneity of the included articles. Fixed-effect model was used for analysis, but if *I*^2^ > 50% or 𝑃 value < 0.05, random effect was applied due to significant heterogeneity [28]. Sensitivity analysis was also done by removing one study from the included studies to testify the stability of results for OS. Egger's funnel regression test and Begg's funnel plot were performed to evaluate the publication, determined as positive by Pr > ∣*z* | ≤0.05.

## 3. Results

### 3.1. Literature Search

**Figure 1:**
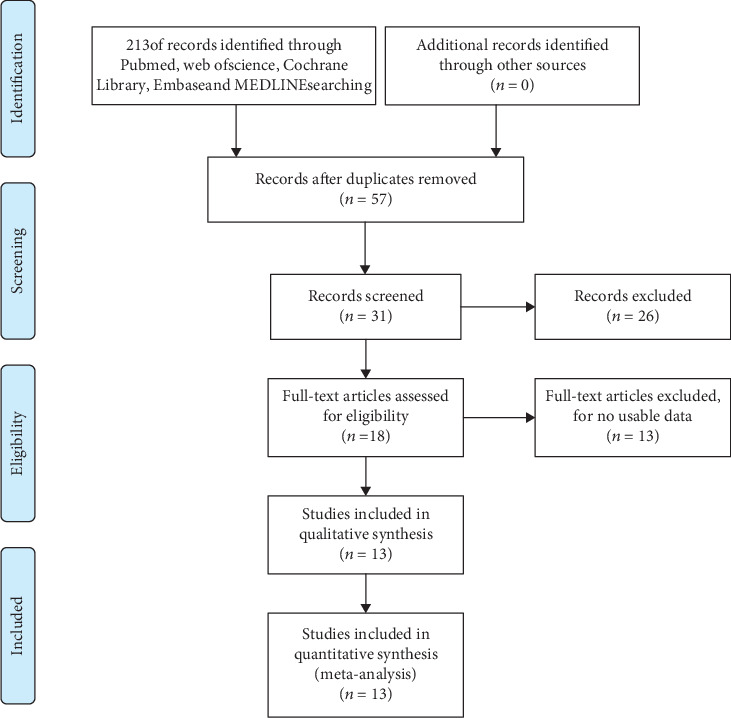
Flow diagram of the literature retrieval and selection.

A total of 213 articles were identified after our preliminary search. Subsequently, 57 studies were left for further inspection after removing duplicate articles. Then, we screened their titles, abstracts, or full texts, and 13 studies were finally included in this meta-analysis [19–23, 29–36]. The procedure of study selection is illustrated in Figure 1.

### 3.2. Characteristics of Included Studies

The main characteristics of the 13 included studies are demonstrated in Table 1. These studies were published between 2016 and 2020, and except one study carried out in Iran, the others were carried out in China. With a total of 919 patients included, the sample size of studies ranged from 32 to 144. These studies reported various cancers, including osteosarcoma (two articles), glioma, ovarian cancer, breast cancer (two articles), acute myeloid leukemia, non-small-cell lung cancer, papillary thyroid carcinoma, gastric cancer, intrahepatic cholangiocarcinoma, and hepatocellular carcinoma (two articles). The level of SNHG3 was quantified with real-time polymerase chain reaction (qRT-PCT). One study measured bone marrow of patients and healthy participants, and the others measured the cancer and matched normal tissues. There were four different cutoff values for dividing patients into high- and low-expression group: five using median, three using mean, one using ROC curve, and three not available. Among these studies, nine studies reported OS, ten studies reported clinical parameters, and one study reported MFS, RFS, and DFS, respectively. With their NOS scores ≥ 7, all included studies showed high quality.

### 3.3. Association between lncRNA SNHG3 Expression and OS

**Figure 2:**
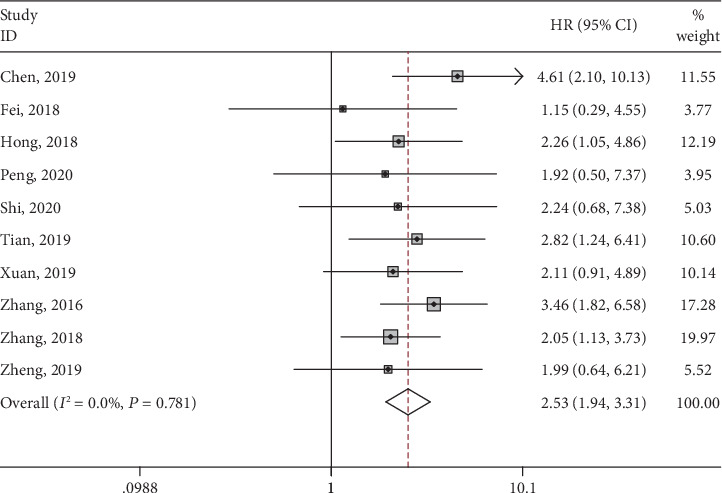
Forest plots of hazard ratios for overall survival in various cancers.

Ten studies reported the relationship between SNHG3 expression and OS, with a total of 737 cases. Fix-effect model was used to analyze the HR and 95% CI of OS due to absence of apparent heterogeneity among included studies (*I*^2^ = 0.0, *P* = 0.781). As shown in Figure 2, the pooled result demonstrated that high expression of SNHG3 was significantly related to poor OS in cancers (HR = 2.53, 95% CI: 1.94-3.31).

**Figure 3:**
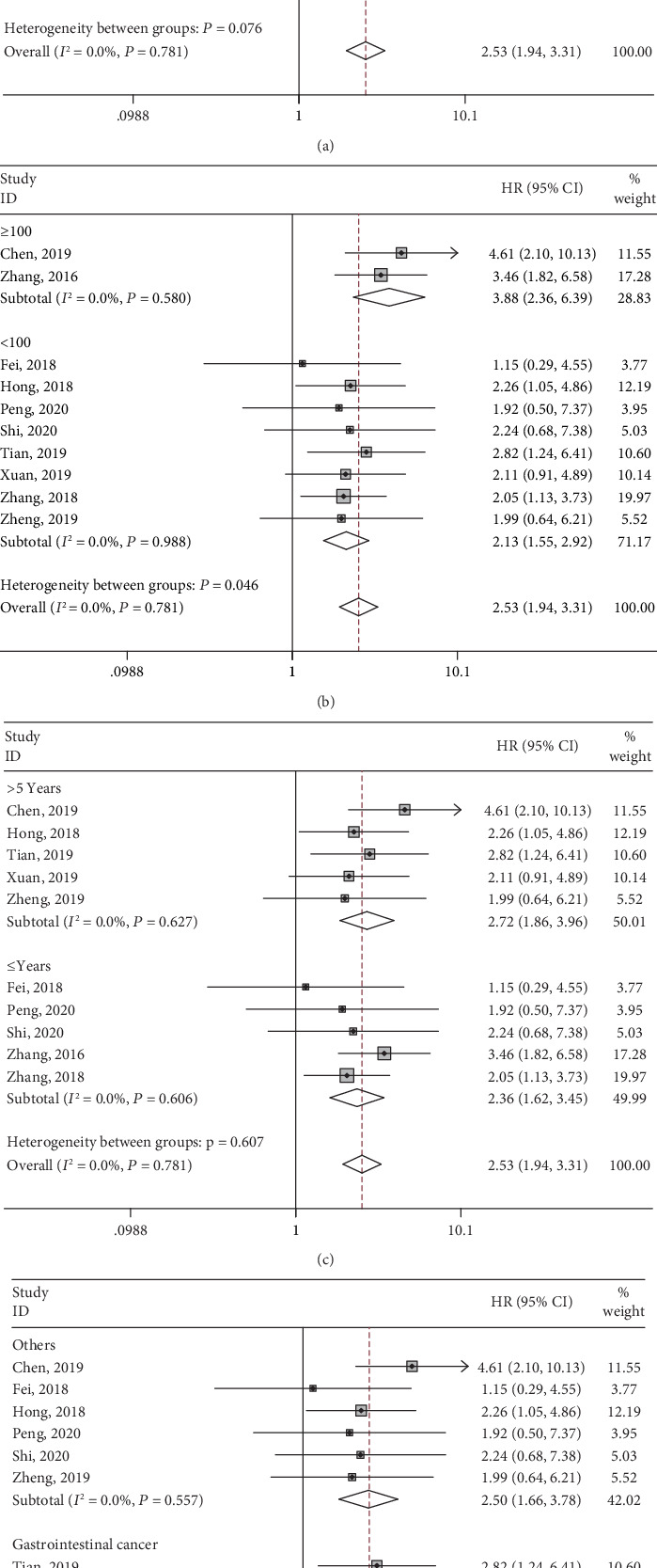
Forest plots of hazard ratios for overall survival: (a) stratified by analysis type, (b) stratified by sample size, (c) stratified by follow-up time, and (d) stratified by cancer type.

To further investigate the relationship between SNHG3 expression and OS, we performed subgroup meta-analysis stratified by analysis method (multivariate and univariate analysis), sample size (more or less than 100), follow-up time (more or less than 5 years), and cancer type (gastrointestinal cancer or others). All subgroup analyses showed similar results that high expression of SNHG3 was significantly associated with worse OS in various cancers (Table 2, Figure 3). All results above demonstrated that SNHG3 could be a prognostic factor for cancer patients' OS.

### 3.4. Association between lncRNA SNHG3 Expression and MFS, DFS, and RFS

Only one study provided suitable data for MFS, DFS, and RFS, respectively. High expression of SNHG3 was significantly correlated to both unfavorable DFS (HR = 3.89, 95% CI: 1.34-11.3), and RFS (HR = 2.42, 95% CI: 1.14-5.15). But it could not predict worse MFS (HR = 1.39, 95% CI: 0.51-3.84). These results indicated that SNHG3 could prompt the prognosis of patients with cancer.

### 3.5. Association between lncRNA SNHG3 and Clinical Parameters

**Figure 4:**
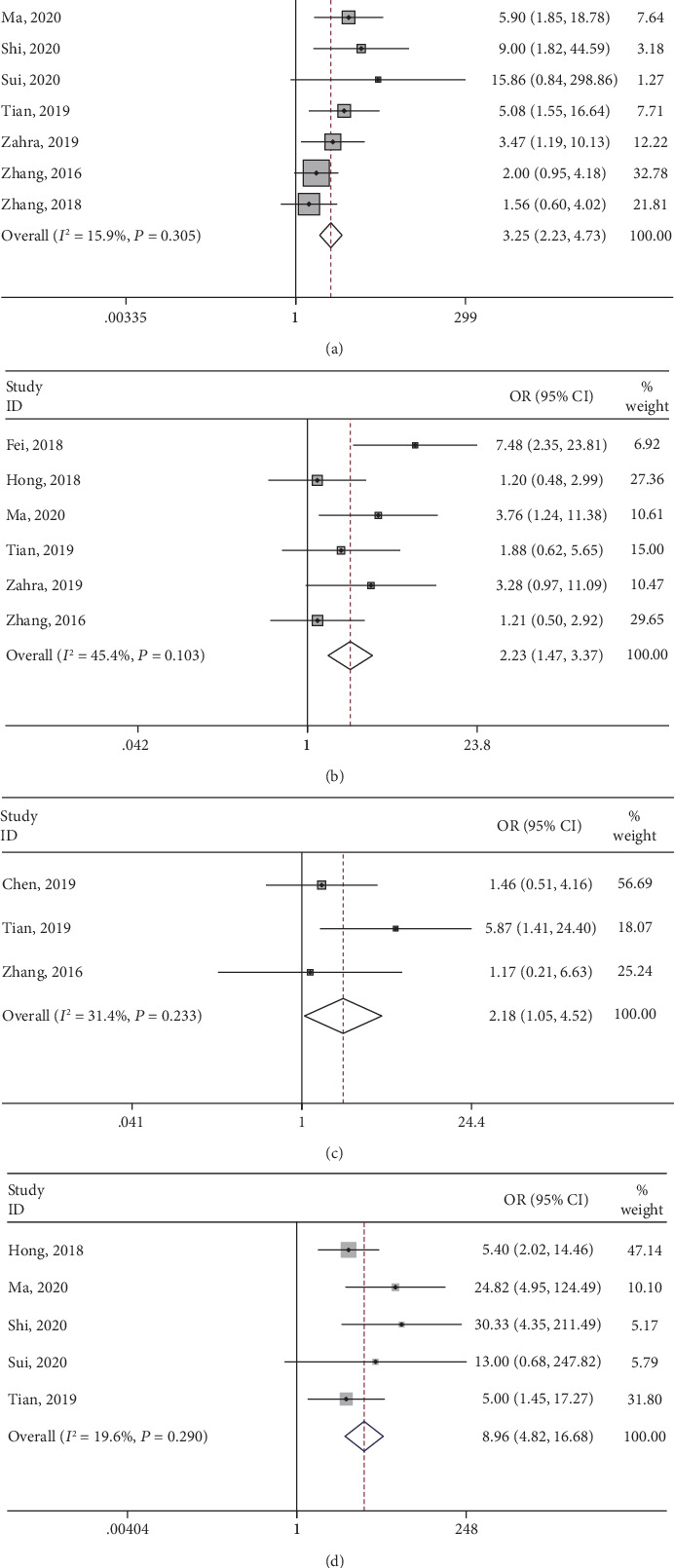
Forest plots of odds ratios for correlation between high SNHG3 expression and clinical parameters: (a) clinical stage, (b) histological grade, (c) distant metastasis, and (d) lymph node metastasis.

Ten studies provided available data for analyses between SNHG3 expression and clinical parameters, including age, gender, clinical stage, tumor size, lymph node metastasis, distant metastasis, and differentiation. ORs and its 95% CIs were adopted for analyses. The results demonstrated that high expression of lncRNA SNHG3 was correlated with later clinical stage (OR = 3.25, 95% CI: 2.23-4.73), higher histological grade (OR = 2.23, 95% CI: 1.47-3.37), distant metastasis (OR = 2.18, 95% CI: 1.05-4.52), and earlier lymph node metastasis (OR = 8.96, 95% CI: 4.82-16.68) (Table 3, Figure 4). Nevertheless, no statistical significance was detected in age (OR = 1.03, 95% CI: 0.74-1.44), gender (OR = 1.06, 95% CI: 0.92-1.21), and tumor size (OR = 1.86, 95% CI: 0.71-4.87) (Table 3).

### 3.6. Sensitivity Analysis

**Figure 5:**
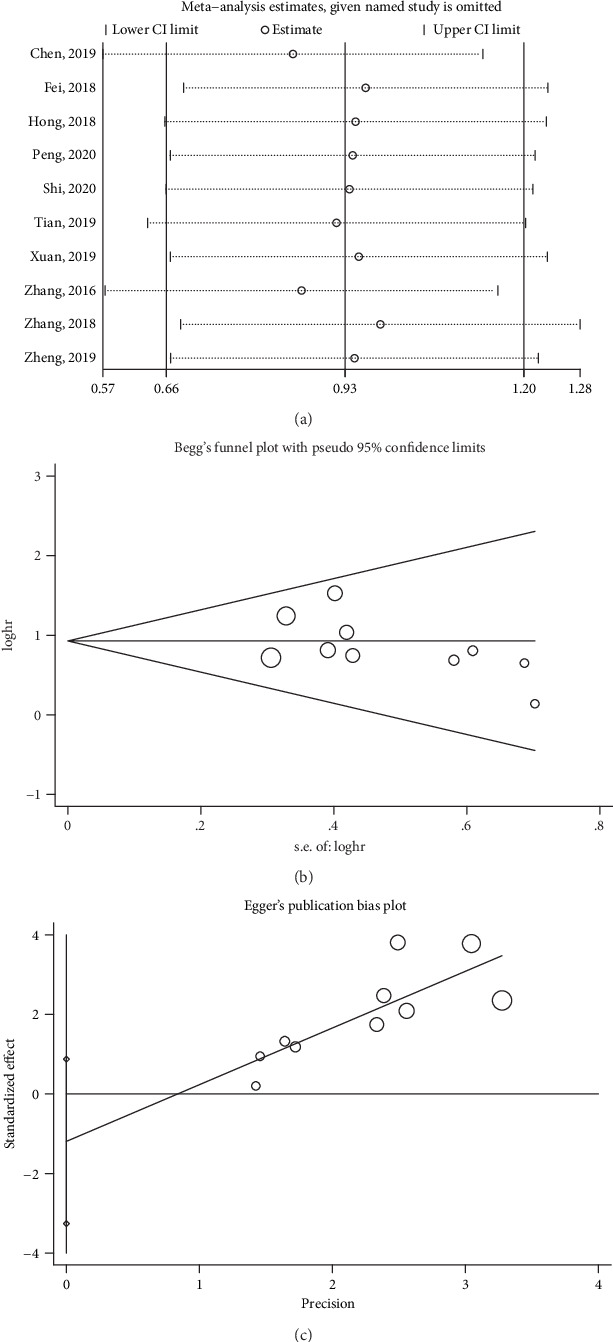
(a) Sensitivity analysis of pooled HRs for OS. (b) Begg's funnel plot of SNHG3 for OS. (c) Egger's funnel plot of SNHG3 for OS.

Then, we executed sensitivity analysis to check the stability of the results of the correlation between SNHG3 expression and OS by removing each study. As shown in Figure 5(a), it was reliable that high expression of SNHG3 was associated with worse OS.

### 3.7. Publication Bias

In addition, we conducted Egger's funnel regression test and Begg's funnel plot to test the publication bias for meta-analysis of the association between SNHG3 expression and OS. There was no significant publication bias based on both Begg's funnel plot (*P* = 0.371, Figure 5(b)) and Egger's funnel regression test (*P* = 0.22, Figure 5(c)), indicating that our pooled result was credible.

### 3.8. Validation of the Role of lncRNA SNHG3 in Human Cancers: Based on TCGA Dataset

**Figure 6:**
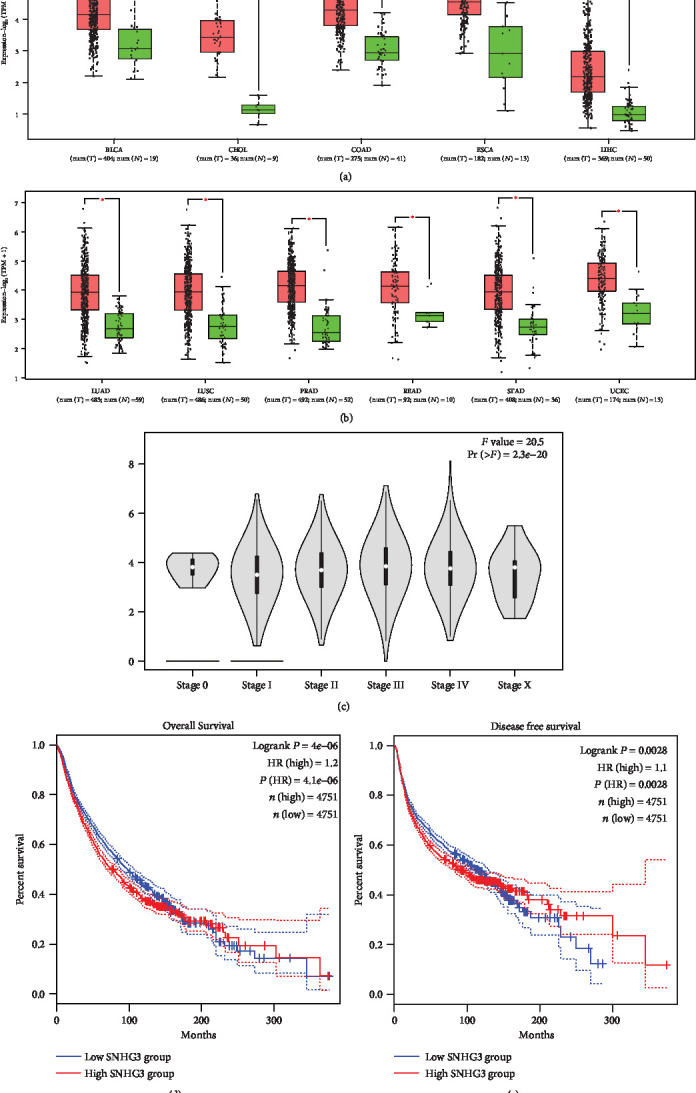
Validation of the role of lncRNA SNHG3 in human cancers in the TCGA dataset: (a) the expression of SNHG3 in cancers and normal tissues, BLCA (bladder urothelial carcinoma), CHOL (cholangiocarcinoma), COAD (colon adenocarcinoma), ESCA (esophageal carcinoma), and LIHC (liver hepatocellular); (b) the expression of SNHG3 in cancers and normal tissues, LUAD (lung adenocarcinoma), LUSC (lung adenocarcinoma), PRAD (prostate adenocarcinoma), READ (rectum adenocarcinoma), STAD (rectum adenocarcinoma), and UCEC (uterine corpus); (c) violin plot of clinical stage of SNHG3 expression in human pancancers; (d) overall survival plot of SNHG3; (e) disease-free survival plot of SNHG3.

To further validate the prognostic and clinical value of SNHG3 expression in human cancers, we investigated TCGA dataset. As shown in Figures 6(a) and 6(b), SNHG3 was significantly overexpressed in tumor tissue comparing to normal tissue in various cancers, including bladder urothelial carcinoma, cholangiocarcinoma, colon adenocarcinoma, esophageal carcinoma, liver hepatocellular, lung adenocarcinoma, lung squamous cell carcinoma, prostate adenocarcinoma, rectum adenocarcinoma, stomach adenocarcinoma, and uterine corpus (*P* < 0.01). Moreover, the violin plot demonstrated that SNHG3 expression was also significantly associated with human pancancers' clinical stage (*P* < 0.01, Figure 6(c)). In addition, the patients were divided into high- and low-SNHG3-expression group according to the median value of its expression. Survival plot was implemented for OS and DFS. The results have shown that high expression of SNHG3 was significantly associated with worse OS (HR = 1.2, *P* < 0.01; Figure 6(d)) and DFS (HR = 1.1, *P* = 0.0028; Figure 6(e)), which ulteriorly confirmed the results of this meta-analysis.

## 4. Discussion

With the development of second-generation sequencing technology in recent years, studies have shown that lncRNA plays an important role in many biological fields such as tumor development, neuroscience, and ontogeny and is an important regulatory molecule in human genome [37, 38]. Increasing studies demonstrate that lncRNAs are dysregulated in cancers and play an important role in tumor development and progression [39, 40]. For example, lncRNA MALAT1 is upregulated in various cancers. High expression of MALAT1 is associated with poor prognosis of cancer patients, and closely correlated with tumor proliferation, autophagy, and drug resistance [41–43].

Studies have demonstrated that SNHG3 is overexpressed in various cancers and considered to function as a novel oncogene in tumor development. Compared to matched normal tissue, SNHG3 is upregulated in tumor tissues, such as osteosarcoma [19, 36], glioma [20], ovarian cancer [21], breast cancer [22, 34], hepatocellular carcinoma [23, 24, 35], acute myeloid leukemia [29], non-small-cell lung cancer [30], papillary thyroid carcinoma [31], intrahepatic cholangiocarcinoma [32], and gastric cancer [33], and usually brings poor clinical outcomes, including OS, DFS, RFS, stage, grade, tumor size, distant metastasis, and lymph node metastasis. What is more, it was found that the level of SNHG3 is significantly associated with epidermal growth factor receptor 2 (Her-2) and estrogen receptor (ER) status in breast cancer [22, 34]. In acute myeloid leukemia, SNHG3 expression was found to have strong association with platelet count and white blood cell count [29].

In our meta-analysis, we explored the relationship between SNHG3 expression and patients' prognosis and other clinical parameters. The results demonstrated that overexpressed SNHG3 was significantly associated with poor OS in various cancers (HR = 2.53, 95% CI: 1.94-3.31) and poor DFS (HR = 3.89, 95% CI: 1.34-11.3) and RFS (HR = 2.42, 95% CI: 1.14-5.15) in hepatocellular carcinoma. Additionally, patients with high SNHG3 expression were more prone to have more advanced clinical stage, higher histological grade, earlier distant metastasis, and earlier lymph node metastasis. In brief, SNHG3 could be a promising biomarker for prognosis in pancancers.

Besides, SNHG3 affects tumorigenesis and prognosis of cancers by participating in various biological process, including promoting cell proliferation, tumor migration and invasion, cell cycle progression, inhibiting apoptosis, and drug resistance [23, 24, 44–47]. Furthermore, many mechanism studies demonstrated that SNHG3 can regulate target genes by miRNA sponge, including miRNA-326 and miRNA-128 in hepatocellular carcinoma [23, 24], miRNA-139-5p in clear cell renal cell carcinoma [44], miRNA-384 in laryngeal carcinoma [45], miRNA-151a-3p and miRNA-196a-5p in osteosarcoma [19, 39], miRNA-758-3p in acute myeloid leukemia [29], and miRNA-182-5p in colorectal cancer [47]. In addition, it could interact with the TGF-*β* and JAK2/STAT3 pathway in lung cancer [30]. Taken together, SNHG3 involved in many aspects of tumor development and it may serve as a promising therapy target.

There are some limits that exist in this meta-analysis. Firstly, most of the enrolled studies were carried out in China with a relatively small sample, which makes the results more suitable in the Chinese population. Secondly, the cutoff values of SNHG3 expression varied from each study and the actual values could not be obtained, which might bring publication bias. Thirdly, the HR value used for analysis was extracted from the K-M curve in some articles, which inevitably brings some bias. Finally, there may exist small bias because we have not yet registered the present study on the PROSPERO Network. Nevertheless, to our knowledge, this is the first paper to comprehensively study the association between SNHG3 expression and clinical outcomes in pancancers. Then, the implementation methods and results recorded in this study strictly complied with the PRISMA statement. At last, almost all analysis used a fixed model which makes the results more credible.

## 5. Conclusion

This study demonstrated that overexpressed SNHG3 was significantly associated with poor survival, including OS, DFS, and RFS and worse clinical outcomes, including clinical stage, histological grade, distant metastasis, and lymph node metastasis in human cancers. Therefore, SNHG3 can serve as a promising biomarker for predicting patients' prognosis.

## Figures and Tables

**Table 1:** The main information of enrolled studies in the meta-analysis.

Study	Year	Country	Tumor types	Sample size	Sample	Detection method	Cutoff value	Follow-up (year)	Outcome measures	Analysis type	NOS
Chen	2019	China	Osteosarcoma	127	Tissues	qRT-PCR	ROC curve	16 years	OS CP	Multivariate	9
Fei	2018	China	Glioma	60	Tissues	qRT-PCR	Mean	5 years	OS CP	Univariate	8
Hong	2018	China	Ovarian cancer	76	Tissues	qRT-PCR	Median	6 years	OS CP	Multivariate	9
Ma	2020	China	Breast cancer	60	Tissues	qRT-PCR	Mean	NA	CP	NA	7
Peng	2020	China	Acute myeloid leukemia	62	Bone marrow	qRT-PCR	Median	5 years	OS CP	Univariate	8
Shi	2020	China	Non-small cell lung cancer	32	Tissues	qRT-PCR	Median	5 years	OS CP	Univariate	9
Sui	2020	China	Papillary thyroid carcinoma	42	Tissues	qRT-PCR	Mean	NA	CP	NA	8
Tian	2019	China	Intrahepatic cholangiocarcinoma	52	Tissues	qRT-PCR	NA	5 years	OS CP	Multivariate	8
Xuan	2019	China	Gastric cancer	60	Tissues	qRT-PCR	Median	6 years	OS MFS	Univariate	8
Zahra	2019	Iran	Breast cancer	80	Tissues	qRT-PCR	NA	NA	CP	NA	7
Zhang	2016	China	Hepatocellular carcinoma	144	Tissues	qRT-PCR	NA	5 years	OS RFS DFS CP	Multivariate	8
Zhang	2018	China	Hepatocellular carcinoma	70	Tissues	qRT-PCR	NA	2 years	OS CP	Univariate	8
Zheng	2019	China	Osteosarcoma	54	Tissues	qRT-PCR	Median	10 years	OS	Univariate	9

**Table 2:** Stratified analyses of overall survival.

Subgroup analysis	No. of studies	No. of patients	HR	95% CL	*I* ^2^(%)	*P*	Model
Analysis method							
Multivariate	4	399	3.20	2.20-4.64	0.0	0.622	Fixed
Univariate	6	338	1.97	1.34-2.9	0.0	0.984	Fixed
Sample size							
≥100	2	271	3.88	2.36-6.39	0.0	0.58	Fixed
<100	8	466	2.13	1.55-2.92	0.0	0.988	Fixed
Follow-up time							
>5 years	5	369	2.72	1.86-3.96	0.0	0.627	Fixed
≤5 years	5	368	2.36	1.62-3.45	0.0	0.606	Fixed
Cancer type							
Gastrointestinal cancer	4	326	2.55	1.80-3.63	0.0	0.653	Fixed
Others	6	411	2.5	1.66-3.78	0.0	0.557	Fixed

**Table 3:** Association between overexpression lncRNA SNHG3 and clinical parameters.

Clinicopathologic parameters	No. of studies	No. of patients	OR	95% CL	*I* ^2^(%)	*P*	Model
Age	9	655	1.03	0.74-1.44	0.0	0.545	Fixed
Gender	8	589	1.06	0.92-1.21	0.0	0.686	Fixed
Clinical stage	8	556	3.25	2.23-4.73	15.9	0.305	Fixed
Tumor size	7	553	1.86	0.71-4.87	76.7	0.0	Random
Lymph node metastasis	5	262	8.96	4.82-16.68	19.6	0.29	Fixed
Distant metastasis	3	323	2.18	1.05-4.52	31.4	0.233	Fixed
Histological grade	4	472	2.23	1.47-3.37	45.4	0.103	Fixed

## Data Availability

The data supporting the conclusions of this study are available from the corresponding author upon request.

## Supplementary Materials

Supplementary Materials Table S1: the search syntax is shown.
