# Supplementary material for: Overexpression of lncRNA SNGH3 Predicts Unfavorable Prognosis and Clinical Outcomes in Human Cancers: Evidence from a Meta-Analysis
Source: Biomed Res Int. 2020 Jun 25;2020:7974034. doi: 10.1155/2020/7974034 (PMC7335396; doi:10.1155/2020/7974034)
Supplement: Supplementary Materials — Table S1: the search syntax is shown. [file 7974034.f1.docx]

**Table S1: Search strategy.**

| 1 | Small nucleolar RNA host gene 3 |
| --- | --- |
| 2 | SNHG3 |
| 3 | #1 or # 2 |
| 4 | neoplasm |
| 5 | cancer |
| 6 | carcinoma |
| 7 | tumor |
| 8 | # 4 or # 5 or # 6 or # 7 |
| 9 | prognosis |
| 10 | Clinical outcome |
| 11 | Pathological feature |
| 12 | survival |
| 13 | # 9 or # 10 or # 11 or # 12 |
| 14 | # 3 and # 8 and #13 |
